# Impact of the Choice of Normalization Method on Molecular Cancer Class Discovery Using Nonnegative Matrix Factorization

**DOI:** 10.1371/journal.pone.0164880

**Published:** 2016-10-14

**Authors:** Haixuan Yang, Cathal Seoighe

**Affiliations:** School of Mathematics, Statistics and Applied Mathematics, National University of Ireland, Galway, Ireland; University of North Carolina at Chapel Hill School of Medicine, UNITED STATES

## Abstract

Nonnegative Matrix Factorization (NMF) has proved to be an effective method for unsupervised clustering analysis of gene expression data. By the nonnegativity constraint, NMF provides a decomposition of the data matrix into two matrices that have been used for clustering analysis. However, the decomposition is not unique. This allows different clustering results to be obtained, resulting in different interpretations of the decomposition. To alleviate this problem, some existing methods directly enforce uniqueness to some extent by adding regularization terms in the NMF objective function. Alternatively, various normalization methods have been applied to the factor matrices; however, the effects of the choice of normalization have not been carefully investigated. Here we investigate the performance of NMF for the task of cancer class discovery, under a wide range of normalization choices. After extensive evaluations, we observe that the maximum norm showed the best performance, although the maximum norm has not previously been used for NMF. Matlab codes are freely available from: http://maths.nuigalway.ie/~haixuanyang/pNMF/pNMF.htm.

## Introduction

Accurate clustering of tumor samples with unknown cancer types or subtypes is of great importance for advances in cancer treatment and for better understanding of biological processes and the mechanisms of cancers. Traditionally, tumors can be clustered into a group based on similar external features such as appearance and clinical evidences. Cancers from within a group discovered by such a procedure may follow significantly different clinical courses and show different responses to therapy [1]. This happens because of lack of internal features revealing intrinsic biological activities. It is therefore desirable to design systematic and objective approaches for recognizing intrinsic tumor types or subtypes based on internal features. With the advent of genomic technologies, the expression levels of thousands genes can be served as internal features, and thus it became possible to design such an approach. This is the objective of molecular cancer class discovery [1, 2]. This is to cluster cancer types based on global gene expression data in an unsupervised setting.

For the task of clustering cancer samples in gene expression data, Nonnegative Matrix Factorization (NMF) [3, 4] has been shown to be a better choice than methods such as hierarchical clustering (HC) or self-organizing maps (SOM) [2]. NMF has several advantages: it is more stable over *a priori* selection of genes in the data or different choices of initial conditions [2]; it explicitly exploits the nonnegativity of the gene expression data; and it does not rely on a similarity metric that may be sensitive to the accumulation of noise in high dimensional data.

Despite its desirable properties, the NMF decomposition is not unique. The NMF algorithm (NMF-Brunnet) [2] does not apply any normalization or regularization methods to NMF solutions. This allows different interpretations of the decomposition and inconsistent clustering results. To alleviate this problem, some methods are proposed that directly enforce uniqueness to some extent by adding regularization terms in the NMF objective function [5–7]. A range of normalization methods have been used: a 2-norm is employed in [5, 7]; 1-norm is used in [3] while the NMF solutions are not normalized in [2, 4, 6].

Here we demonstrate the simple employment of a normalization method without using regularization techniques can achieve promising performance in clustering accuracy. Specifically, we propose to use the maximum norm (infinity norm) to normalize NMF solutions—a normalization method that has not previously been used in clustering analysis using NMF techniques for molecular cancer class discovery—a post-processing method, through which NMF becomes less sensitive to *a priori* selection of genes or initial conditions, and more robust to data variation.

Algorithms for molecular cancer class discovery have to be evaluated using sample labels for cancer types. There are datasets for which blind class discovery based on clustering will not work either because there are other categorical variables (gender, origin of tissues, and so on) that interfere with identification of the clusters of interest, or because there are just too few features that discriminate between the classes. Datasets with such a problem are not appropriate to be employed to compare the performance of different clustering algorithms, despite that they may be good for evaluating classification algorithms. This is because that some classification algorithms can overcome this problem through selecting relevant features by using the sample labels while this is impossible for a clustering algorithm without accessing sample labels.

## Materials and Methods

Gene expression data can be represented as an *n* × *m* matrix *A*, where *n* and *m* are the number of genes and the number of samples respectively. As expression level is nonnegative, here *A* is a nonnegative matrix, where each row represents the expression level of a gene across all samples, and each column the expression level of all genes in one sample.

An NMF method [4] employed in [2] is able to take into account the nonnegative property of the gene expression matrix to achieve better clustering results than SOM and HC. This method resorts to a low-rank approximation of the gene expression matrix *A* by the product of two nonnegative matrices *W* of size *n* × *k* and *H* of size *k* × *m*, *i.e.*, factoring *A* into *W* and *H*, denoted as *A* ∼ *WH*. *k* is a predefined number that is usually much smaller than *m*, and in our context, it is the number of clusters. Each column of *W* defines a metagene, with entry *w*_*ij*_ the coefficient of gene *i* in metagene *j*. The columns of matrix *H* represent the metagene expression pattern of the corresponding samples, with each entry *h*_*ij*_ represent the expression level of metagene *i* in sample *j*.

Given such a factorization, the matrix *H* can be used to determine the cluster membership: sample *j* is placed in cluster *i* if *h*_*ij*_ is the largest entry in column *j* [2]. Thus different *H*s may produce different clustering results. An appropriate normalization of the factor matrices returned by a Basic NMF algorithm is expected to produce an *H* through which better clustering results can be found.

Note that, we are trying to investigate the effect of different normalization methods on NMF clustering as it is currently practiced. The above membership assignment method is what is currently practiced [2, 5–10]. It can be explained as follows. From the approximation *A* ∼ *WH*, the *j*-th column *A*_:,*j*_, representing sample *j*, of *A* can be approximated by *A*_:,*j*_ ∼ ∑_*i*_ *W*_*i*_ *h*_*ij*_, thus *h*_*ij*_ has the meaning of the strength that sample *j* is associated to metagene *i* defined by *W*_*i*_—*i*-th column of *W*. Therefore membership assignment of sample *j* by comparing the values (*h*_*ij*_, *i* = 1, 2, …, *k*) in the *j*-th column of *H* is justified. Thus it is a natural choice to use this membership assignment method for NMF algorithms.

### A Basic NMF Algorithm (NMF-Brunet)

A Basic NMF method is described in [2, 4] where the NMF solutions are not normalized. Given a nonnegative matrix *A* and a desired factorization rank *k*, the objective of the NMF algorithm is to find an approximation *A* ∼ *WH*. After initializing nonnegative matrices *W* and *H* randomly, they are iteratively updated to minimize a loss function *L*(*A*, *WH*), specified as a generalized KL-divergence functional in [2]:

$$L\left(A,WH\right)=\sum_{i,j}A_{i,j}\log\left(A_{i,j}/{\left(WH\right)}_{i,j}\right)-A_{i,j}+{\left(WH\right)}_{i,j}.$$ (1)

In each iteration, *W* and *H* are updated by using the coupled multiplicative update rules:

$$H_{au}\leftarrow H_{au}\frac{\sum_{i}W_{ia}A_{iu}/{\left(WH\right)}_{iu}}{\sum_{k}W_{ka}},W_{ia}\leftarrow W_{ia}\frac{\sum_{u}H_{au}A_{iu}/{\left(WH\right)}_{iu}}{\sum_{v}H_{av}}.$$

By the above update rules, both *W* and *H* preserve the nonnegativity property.

Underlying the generalized KL divergence functional *L*, is a statistical model [3, 11]—*A*_*i*,*j*_ (understood as an integer) is an observation from a Poisson distribution with mean (*WH*)_*i*,*j*_, and *W* and *H* are model parameters. The log-likelihood for a single observation *A*_*i*,*j*_ can be written as − (*WH*)_*i*,*j*_ + *A*_*i*,*j*_ log((*WH*)_*i*,*j*_) − *C* where *C* is a constant with respect to *W* and *H*. The log-likelihood for the overall observation *A* is then *l* = ∑_*i*,*j*_ − (*WH*)_*i*,*j*_ + *A*_*i*,*j*_ log((*WH*)_*i*,*j*_) − *C*. Thus maximizing *l* is equivalent to minimizing *L*. If *A*_*i*,*j*_ is a real nonnegative number, then the above argument can be set for the integer part of *A*_*i*,*j*_, and the resulting log-likelihood is near to *l* = ∑_*i*,*j*_ − (*WH*)_*i*,*j*_ + *A*_*i*,*j*_ log((*WH*)_*i*,*j*_) − *C*. Therefore the Basic NMF algorithm is a Maximum Likelihood Estimation of the model parameters *W* and *H* assuming independent Poisson distributions for all entries in *A*. This is quite reasonable for nonnegative data [11]. Moreover, if, for a constant *c* ≠ 1, for all entries in *A*, *cA*_*i*,*j*_ is (*A*_*i*,*j*_ itself is not) an observation from a Poisson distribution with mean *c*(*WH*)_*i*,*j*_, minimizing *L* can still be justified by maximizing a corresponding log likelihood. Thus, minimizing Eq (1) is supported by both Poisson noises or scaled Poisson noises generating *A*, under the Maximum Likelihood Principle.

### The post-processing method

It is well-known that the decomposition of *A* ∼ *WH* is not unique, and an NMF algorithm may not converge to the same solution on each run, depending on the random initial conditions. As explained in [2] the above NMF algorithm converges towards a relatively fixed attractor irrespective of random initial conditions if a clustering into *k* classes is strong. In that case, we would expect that sample assignment to clusters would vary little from run to run. However, this stability of the NMF algorithm is still not very satisfactory as it does not solve the non-uniqueness problem. For example, if *A* ∼ *WH* is a solution returned by an algorithm, *A* ∼ *WD*^−1^ *DH* = *W*′ *H*′ is an equivalent solution where *W*′ = *WD*^−1^, *H*′ = *DH*, and *D* is any positive diagonal matrix of order *k*. But *H* and *H*′ may produce different clustering results.

The idea of this paper is that, among all possible (infinitely many) such solutions, we prefer the *W*′ whose columns are standardized so that the entries in each column of *H*′ (metagene expression pattern of the corresponding sample) are more comparable, resulting in a more reasonable cluster membership assignment. In addition, since only one solution is chosen among infinitely many equivalent solutions, the non-uniqueness problem of the NMF solution becomes alleviated, resulting in a more stable cluster membership assignment.

In different contexts, standardization of *W* may have different mathematical forms. Here we explore several alternative forms for the standardization for *W*, empirically identifying the optimal choice based on several independent datasets.

#### Normalization methods

Some quantities of columns of *W* such as various sample quantiles (0.95 quantile, 0.75 quantile, and 0.5 quantile), the sample standard deviation, p-norms (1-norm, 2-norm, 3-norm, and the infinity norm) can be used as the basis of metagene normalization. Note that the infinity norm is also called the maximum norm. Taking the maximum norm as an example, the post-processing method simply standardizes each column of *W* by dividing by its maximum, which is used to multiply the corresponding row of *H*. More generally, given a specific norm or a function that maps a vector to a real value (for example, the maximum norm maps a vector to its maximum value), the post-processing method is described in a matrix form as follows. Let *A* ∼ *WH* be a solution returned by a Basic NMF algorithm. Let *D* be a diagonal matrix whose *j*-th diagonal entry is *D*_*j*_ that is mapped from the *j*-th column of *W*. The standardized solution is thus *W*′ = *WD*^−1^, *H*′ = *DH*. It is easy to implement: *H*′ is obtained by multiplying each row of *H* by the mapped value of the corresponding column of *W* while columns of *W* are normalized by dividing each column of *W* by its mapped value. Then we use the matrix $H^{\prime }=\left(h_{ij}^{\prime }\right)$ to determine the cluster membership: sample *j* is placed in cluster *i* if the $h_{ij}^{\prime }$ is the largest entry in column *j*. This post-processing method has the advantage that the product of *W*′ and *H*′ is the same as that of *W* and *H*—this does not change the approximation to *A*, and hence does not change the statistical justification underlying the objective function in Eq (1). Note that *W*′ needs not to be calculated if we only need *H*′ to determine the cluster membership in the setting of the problem in this paper. However, *W*′ contains some information that can be employed to construct an embedded filter as shown below.

#### An embedded filter

There may be many factors distorting the ‘right’ NMF solutions, based on which the standardized NMF solutions may not be the right ones. Thus with distorting factors present, it is not easy to draw a consistent conclusion about the optimal choice of a normalization method. However, we can sort out one such factors—the effects from irrelevant genes—by an ad hoc embedded filter.

Irrelevant genes may disturb exact values in *W* and *H* so that the post-processing method which is based on fine-tuning of *W* and *H* may not work properly. To remove a large proportion of irrelevant genes, we add an embedded filter after the algorithm described above, and after this filtering, we then apply the above algorithm again.

Genes (though significantly expressed in *A*) that have similar values across all metagenes in *W*′ after the normalization method are not informative because they are unlikely to distinguish between different typical cancer types. Thus we filter out all those genes for which the difference between maximal value and minimal value in the metagenes does not exceed a threshold—the default is the median of all of such differences. This filter depends on the Basic NMF algorithm and the quality of normalization methods, and we expect that an appropriate normalization method will make columns of *W* more comparable so that this filter will make sense. The post-processing method with/without the embedded filter is summarized in Algorithm 1.

Note that the embedded filter is only an auxiliary tool that helps show the impact of the choice of normalization method. The embedded filter itself is interesting, but ad hoc at the moment as the optimal threshold needs to be determined. Other filters independent of an NMF algorithm may also help remove irrelevant genes. However, they are not specially designed for NMF algorithms, and thus may not be optimal for NMF algorithms. Nevertheless, it is interesting to see the effects of normalization methods if a different filter is used. Thus we replace the embedded filter with a variance-filter [12] for the purpose of extensive evaluations. This simple filter removes genes with low variance across samples, and it does not depend on an NMF algorithm. Similar to the embedded filter, we use this filter to remove half of the genes, run NMF algorithms, and then compare the effects of normalization methods. Different from the embedded filter, we do not need to run NMF twice for the variance-filter.

**Algorithm 1** Post-processing of a Basic_NMF for clustering based on a given normalization method that maps a column of a matrix to a value.

Input: an *n* × *m* matrix *A*; a predefined number of clusters *k*;

a fraction *T* of genes that will be filtered out (default: T = 0.5); and a switch Filter (default: Filter = 1 if use the filter; Filter = 0 otherwise).

Output: a *k* × *m* matrix $H_{2}=\left(h_{ij}^{\prime }\right)$. Note that *H*_2_ can be used to determine the cluster membership: sample *j* is placed in cluster *i* if the $h_{ij}^{\prime }$ is the largest entry in column *j*.

1: Run Basic_NMF on *A*, and get [*W*_0_,*H*_0_]

2: **if** Filter==0 **then**

3:   Obtain *H*_2_ by multiplying each row of *H*_0_ by the normalized value of the corresponding column of *W*_0_

4: **else**

5:   Obtain *W* by dividing each column of *W*_0_ by the normalized value of this column

6:   Generate a vector *u* of size *n* × 1 by calculating the difference between the maximum value and the minimum value for each row of *W*

7:   Choose index set *I* = {*p*: *u*(*p*)≤*T* quantile of *u*}

8:   Get a matrix *A*_1_ by discarding rows of *A* with indices in *I*

9:   Run Basic_NMF on *A*_1_, and get [*W*_1_,*H*_1_]

10:  Obtain *H*_2_ by multiplying each row of *H*_1_ by the normalized value of the corresponding column of *W*_1_

11: **end if**

#### Selection of *k*

As we shall observe (see Results), using the maximum norm is the best choice. Accompanying the proposed normalization method, we also propose the corresponding cophenetic correlation coefficient to measure the stability for each value of *k*, and this measure can be used to select an appropriate *k*. Our method is a straightforward adaption of that in [2]. This method is described in Algorithm 2.

The justification of using the cophenetic correlation coefficient as a measure for model selection is made in [2]: if *k* is the true number of clusters, a clustering into *k* classes should be strong, and we would expect that sample assignment to clusters would vary little from run to run; consequently the entries in the consensus matrix (the average connectivity matrix over many clustering runs) should be close to 0 or 1; the cophenetic correlation coefficient of the consensus matrix can then be used as a measure of the robustness of the class assignments with respect to random initial conditions; thus a larger cophenetic correlation coefficient for *k* provides evidence that *k* is a better choice.

While we inherit this justification, we argue that, for a given *k*, the variation of sample assignment to clusters depends on two factors: the initial conditions and normalization methods. The choice of the normalization methods is a nuisance factor, and should be removed as the post-processing method suggests. After the post-processing method, there is one factor left that we need to consider: the initial conditions are randomly generated as [2]. The post-processing method removes the non-uniqueness of a given decomposition *A* ∼ *WH* to some extent, resulting in more stable cluster membership. The remaining variation in the cluster assignment captured by the cophenetic correlation coefficient in Algorithm 2 will then reflect the stability of the cluster assignment with respect to the initial conditions, and may thus provide a better means to identify *k*.

**Algorithm 2** Cophenetic correlation coefficient. Input: an *n* × *m* matrix *A*; a predefined number of clusters *k*; a predefined number of loops nloop. Output: Cophenetic correlation coefficient *r*.

1: Run the steps 2–4 nloop times

2: Run Basic_NMF on *A* with the initial conditions randomly generated, and get [*W*, *H*]

3: Obtain *H*_1_ by multiplying each row of *H* by the maximum value of the corresponding column of *W* {because the maximum norm will be shown to be the best choice as a normalization method}

4: Determine the cluster membership by $H_{1}=\left(h_{ij}^{\prime }\right)$: sample *j* is placed in cluster *i* if the $h_{ij}^{\prime }$ is the largest entry in column *j*

5: Calculate the consensus matrix $\bar{C}$ and Pearson correlation *r* of two distance matrices as in [2]: the first, $1-\bar{C}$, is the distance between samples induced by the consensus matrix, and the second is the distance between samples induced by the linkage used in the reordering of $\bar{C}$.

### Evaluation

In order to evaluate the effect of different normalization methods with/without the filter in Algorithm 1, we need to compare their performance in terms of clustering accuracy on some datasets that should be selected in a fair and unbiased manner. For this purpose, we need to choose datasets carefully. Our strategy is to use some well-studied datasets as the core datasets so that it is appropriate to do extensive analyses on them as information about true number of clusters, data quality, and results obtained by others is well known. We expect that the conclusions drawn from the core datasets are more convincing than those from other datasets less studied. Nevertheless, evaluation on more datasets helps draw conclusions with more confidence, so we also choose some datasets from GEO. In addition, we select a MicroRNA expression data used in [8] because NMF-Brunet was employed for clustering analysis on this dataset.

### Datasets

Datasets selected are described below. A brief description of these datasets is reported in Table 1.

**Table 1:** Dataset description and performance comparison in term of clustering accuracy in percentage. Reported is the mean of clustering accuracies from 100 runs of Basic NMF together with the standard error of the mean. Also reported is the p-value produced by a paired two-sided t-test. Note that the proposed method is using ‘max’ normalization and using the filter.

Datasets	#Types	#Samples	#Genes	Basic NMF (NMF-Brunet)	Post-Processing	p-values
**Core Datasets**						
Leukemia	2	38	5,000	94.95±0.10	**100±0**	6.70e-074
Leukemia	3	38	5,000	95.58±0.14	**100±0**	2.56e-052
CNS	4	34	6,881	94.03±0.05	**96.88±0.07**	2.02e-054
Medulloblastoma	2	34	5,893	59.59±0.13	**61.76±0**	1.02e-030
**Extended Datasets**						
Breast Recurrence (GSE4913)	2	50	5,000	54.32 ±0.14	**62.48±0.28**	2.61e-045
Pancreas (GSE46385)	5	47	5,000	46.04±0.51	**51.57±0.60**	9.43e-011
Colorectal (GSE35896)	2	62	5,000	87.11±0.40	**88.00±0.32**	0.069
Colorectal (GSE42284)	4	188	5,000	**33.00±0.10**	32.13±0.12	1.66e-007
Breast_MicroRNA (PAM50)	5	503	1,007	36.10±0.06	**45.12±0.02**	6.78e-120
Breast MicroRNA (Histologic)	4	643	1,017	46.73±0.20	**74.24±0.13**	6.59e-107
Breast MicroRNA (ER)	2	677	1,022	58.30±0.35	**73.34±0.11**	4.94e-066
Breast MicroRNA (PR)	2	674	1,022	56.31±0.35	**65.93±0.11**	9.10e-047
Breast MicroRNA (HER2)	2	487	1,009	58.50±0.04	**69.99±0.03**	5.90e-141

#### Core datasets

We start the evaluation on three gene expression datasets: Leukemia dataset [1], Central Nervous System tumors (CNS) [13], and Medulloblastoma dataset [13]—these are our core datasets because they are well-studied—they were commonly used by [2, 5–7, 9] as their all test cases for the purpose of comparing the performance of different clustering algorithms. Moreover, in the seminal study [1], the Leukemia dataset is used as the only test case; in the review paper [10], it is used as the only illustrative example; and in [14] it is used as the only biological example. Below is a brief description of these three datasets.

- Leukemia dataset It is known that Acute Myelogenous Leukemia (AML) and Acute Lymphoblastic Leukemia (ALL) are distinct, and ALL can be further divided into two subtypes: T and B [1]. Here we used the same data as [2] that contains the 5,000 most highly varying genes. The dataset contains 38 samples. The Leukemia dataset is well established and has served as a benchmark dataset for comparing the performance of different clustering algorithms. In testing clustering algorithms, there are two settings for this dataset: two types of cancers and three types of cancers. If it is considered to contain two types of cancers, the task is to distinguish AML and ALL which is relatively easy. For the setting of three types of cancers, in addition, ALL can be further divided into T and B subtypes, thus there are three types: AML, ALL-T and ALL-B. Note that 38 bone marrow samples can be assigned to the aforementioned three subtypes with high confidence based on clinical and histopathological evidences.
- Central nervous system tumors (CNS) This dataset contains 34 samples and is composed of four types of central nervous system embryonal tumors: 10 classic medulloblastomas, 10 malignant gliomas, 10 rhabdoids and 4 normals [2].
- Medulloblastoma dataset Medulloblastoma is a childhood brain tumor and the pathogenesis of these tumors is not well understood [2, 5]. This dataset contains 34 samples, consisting of two types of tumor from the point of view of histological subclasses: 25 classic and 9 desmoplastic medulloblastomas.

#### Extended datasets

Besides the above core datasets, we extend the evaluation to four additional datasets. We selected them from GEO Datasets by searching keywords “clustering”, “Homo sapiens”, and “Cancer”. Since the number of samples are too few, we skipped the datasets GSE73099 (2 samples), GSE66733 (2 samples), GSE67939 (17 samples), GSE65201 (8 samples), GSE60271 (14 samples), GSE58478 (4 samples), GSE55807 (6 samples), GSE58326 (6 samples), GSE44800 (8 samples), and GSE32132 (8 samples). Since the research is mainly focused on supervised learning by selecting a small set of signature genes, we skipped the datasets GSE69259, GSE51827, GSE67994, GSE68545, and GSE68104. The reason for this can be found in the discussion on evaluation of unsupervised methods using datasets designed for supervised algorithms in Section Discussion. Since the data has not clear cancer types, we skipped the datasets GSE70233, GSE68606, GSE51334, and GSE60272. We skipped some more datasets because that there is no gene expression data (GSE60270), the study is complicated (GSE55689), or there is no difference between the Basic NMF and the normalized one (GSE45419).

We finally chose 4 datasets from GEO: Breast carcinomas and local recurrence (GSE4913) [15], pancreatic Cancers (GSE46385) [16], Colorectal Cancers (GSE35896) [17], and GSE42284 [18]. These datasets are described below. For these four datasets, we used the 5,000 most highly varying genes.

- GSE4913 This dataset is for the analysis of primary breast carcinoma tumors from 50 patients who received breast-conserving therapy (BCT). There are two subtypes: 19 patients subsequently developed a local recurrence of the carcinoma while 31 did not.
- GSE46385 There are 47 samples in this dataset. There are 5 types: Patient pancreas tissue, Patient pancreatic tumor, Patient-derived cancer cell line, Patient-derived cancer cell line in mouse, and Patient-derived tumor in mouse.
- GSE35896 This gene expression data is from 62 colorectal cancers. There are two dominant CRC subtypes that were discovered by a pathway-based stratification of CRC tumor samples.
- GSE42284 This gene expression data is from 188 primary colorectal cancers with four cancers stages.

#### MicroRNA expression for Breast cancer

Normalized read count data for 697 tumor samples were extracted from Level 3 data archives on the TCGA Data Portal website. The most variable 25% microRNAs were used as input to an NMF algorithm. For more details, see the supplementary materials of [8]. We used clinical categorical features to evaluate the effectiveness of the clustering algorithm. We used the following available clinical information to evaluate subtype identification results: estrogen receptor (ER) status (positive or negative); progesterone receptor (PR) status (positive or negative); her2/neu immunohistochemistry receptor (HER2) status (positive or negative); and optical measurement histologic type (Histologic) (Infiltrating Ductal, Infiltrating Lobular, Medullary, Mucinous). In addition, we also used the sample assignment determined by the 50-gene PAM50 model [19] which has five subtypes (‘Basal’,‘Her2’,‘LumA’,‘LumB’,‘Normal’). For each categorical feature, we removed the samples whose statuses are ambiguous. This procedure results in different sample sizes for different clinical features. These statuses are: ‘Indeterminate’, ‘Not Performed’, ‘Performed but Not Available’, ‘Mixed Histology’, and ‘Other’. In addition, we removed the genes whose values are all zeros for all the remaining samples in each clinical feature. This results in different sets of genes for different clinical features.

## Results

We show the results in Table 2 for three settings: using the embedded filter, or using the variance-filter, or not using a filter. For both filters, we removed half of the genes—this amounts to using the default T = 0.5 for the embedded filter. By randomly selecting the initial *H* and *W*, we performed 100 independent trials and computed the mean accuracy and the standard error of the mean. Note that the Basic NMF (here it is the NMF-Brunet) does not normalize the NMF solutions and it does not use the filter in Algorithm 1.

**Table 2:** Comparison of different normalization methods in term of clustering accuracy in percentage. Reported is the mean of clustering accuracies from 100 runs of Basic NMF together with the standard error of the mean.

Datasets	Unnormalized	0.95q	0.75q	0.5q	SD	1-norm	2-norm	3-norm	max
**Using the embedded filter** (Filter = 1 in Algorithm 1)									
Leukemia (k = 2)	95.08±0.12	**100±0**	97.37±0	97.37±0	97.37±0	97.37±0	**100±0**	**100±0**	**100±0**
Leukemia (k = 3)	95.87±0.15	97.37±0	94.74±0	96.82±0.11	97.37±0	97.37±0	97.37±0	97.37±0	**100±0**
CNS	93.35±0.28	91.18±0	94.03±0.07	94.12±0	93.82±0.27	94.09±0.03	93.82±0.29	94.12±0	**96.88±0.07**
Medulloblastoma	60.24±0.15	55.88±0	**61.76±0**	**61.76±0**	58.82±0	**61.76±0**	58.82±0	60.00 ±0.14	**61.76±0**
Breast Recurrence (GSE4913)	54.76±0.12	56.22±0.07	54.00±0	54.00±0	54.00±0	54.00±0	54.00±0	54.00±0	**62.48±0.28**
Pancreas (GSE46385)	46.68±0.52	49.53±0.54	48.21±0.57	47.00±0.63	50.85±0.55	48.43±0.54	48.85±0.53	49.06±0.57	**51.57±0.60**
Colorectal (GSE35896)	**88.44±0.31**	85.37±0.36	86.42±0.39	87.45±0.36	80.74±0.25	86.85±0.40	87.74±0.34	86.60±0.36	88.00±0.32
Colorectal (GSE42284)	32.38±0.11	31.28±0.10	32.05±0.16	**33.97±0.18**	31.68±0.13	33.51±0.16)	32.66±0.14	32.03±0.13	32.13±0.12
Breast MicroRNA (PAM50)	36.01±0.09	39.08±0.09	34.95±0.06	36.08±0.09	42.25±0.06	35.49±0.05	42.31±0.06	43.18±0.07	**45.12±0.02**
Breast MicroRNA (Histologic)	46.71±0.18	55.88±0.61	57.27±0.16	59.78±0.70	69.99±0.05	60.25±0.22	70.00±0.06	72.08±0.08	**74.24±0.13**
Breast MicroRNA (ER)	58.00±0.33	69.09±0.13	65.85±0.11	65.00±0.17	68.58±0.22	63.65±0.30	68.89±0.23	71.01±0.16	**73.34±0.11**
Breast MicroRNA (PR)	56.47±0.33	63.51±0.14	61.39±0.13	61.07±0.08	63.44±0.23	59.58±0.29	63.27±0.21	64.31±0.13	**65.93±0.11**
Breast MicroRNA (HER2)	58.46±0.04	61.55±0.04	59.57±0.04	55.48±0.10	66.01±0.02	59.94±0.02	65.92±0.03	67.55±0.03	**69.99±0.03**
**Without Filter**(Filter = 0 in Algorithm 1)									
Datasets	**NMF-Brunet**	0.95q	0.75q	0.5q	SD	1-norm	2-norm	3-norm	max
Leukemia (k = 2)	94.95±0.10	97.37±0	97.37±0	97.37±0	**100±0**	97.37±0	**100±0**	**100±0**	**100±0**
Leukemia (k = 3)	95.58±0.14	97.37±0	94.74±0	94.74±0	97.37±0	97.37±0	97.37±0	97.37±0	**100±0**
CNS	94.03±0.05	93.85±0.08	**94.12±0**	**94.12±0**	**94.12±0**	**94.12±0**	**94.12±0**	**94.12±0**	91.26±0.07
Medulloblastoma	59.59±0.13	58.82±0	**61.76±0**	**61.76±0**	55.88±0	**61.76**±0	56.35±0.11	58.76±0.04	61.53±0.08
Breast Recurrence (GSE4913)	54.32±0.14	54.00±0	54.68±0.17	**56.64±0.17**	54.00±0	53.88±0.13	53.60±0.10	54.00±0	50.00±0
Pancreas (GSE46385)	46.04±0.51	48.91±0.59	48.38±0.62	47.72±0.63	50.19±0.56	48.13±0.61	48.60±0.59	48.81±0.58	**50.79±0.57**
Colorectal (GSE35896)	87.11±0.40	86.85±0.36	86.87±0.37	86.06±0.44	85.06±0.33	86.61±0.40	86.69±0.39	86.73±0.38	**88.92±0.29**
Colorectal (GSE42284)	33.00±0.10	32.11±0.13	34.20±0.13	**36.02±0.20**	31.58±0.10	34.52±0.15	33.63±0.13	33.13±0.12	32.46±0.14
Breast MicroRNA (PAM50)	36.10±0.06	36.01±0.02	36.00±0.08	31.41±0.04	42.46±0.06	35.58±0.05	42.45±0.06	43.27±0.02	**45.12±0.02**
Breast MicroRNA (Histologic)	46.73±0.20	55.19±0.28	61.25±0.65	67.16±0.67	69.92±0.05	60.01±0.25	69.90±0.05	72.07±0.06	**74.11±0.11**
Breast MicroRNA (ER)	58.30±0.35	65.53±0.12	64.75±0.13	66.26±0.28	68.86±0.23	63.58±0.31	68.85±0.23	70.91±0.15	**73.24±0.11**
Breast MicroRNA (PR)	56.31±0.35	61.53±0.13	60.90±0.08	61.95±0.12	62.94±0.21	59.74±0.30	62.95±0.21	64.25±0.12	**65.85±0.10**
Breast MicroRNA (HER2)	58.50±0.04	58.85±0.04	54.33±0.05	53.14±0.09	65.94±0.02	59.94±0.02	65.88±0.03	67.53±0.04	**69.97±0.03**
**Using the variance-filter**									
Datasets	**NMF-Brunet**	0.95q	0.75q	0.5q	SD	1-norm	2-norm	3-norm	max
Leukemia (k = 2)	94.97±0.09	**100±0**	97.37±0	97.37±0	**100±0**	97.37±0	**100±0**	**100±0**	**100±0**
Leukemia (k = 3)	95.61±0.12	97.37±0	94.74±0	94.74±0	97.37±0	97.37±0	97.37±0	97.37±0	**100±0**
CNS	92.09±0.15	91.09±0.05	93.53±0.12	93.09±0.14	**94.06±0.04**	94.03±0.05	**94.06±0.04**	**94.06±0.04**	93.62±0.11
Medulloblastoma	61.35±0.10	58.82±0	**61.76±0**	**61.76±0**	58.82±0	**61.76±0**	61.71±0.04	**61.76±0**	**61.76± 0.00**
Breast Recurrence (GSE4913)	**71.50±0.11**	70.24±0.07	70.24±0.07	70.24±0.07	68.48±0.13	70.24±0.07	70.24±0.07	70.24±0.07	57.50±0.54
Pancreas (GSE46385)	45.53±0.45	46.74±0.50	46.62±0.53	47.30±0.55	46.87±0.56	47.13±0.53	47.02±0.53	46.83±0.52	**48.02±0.52**
Colorectal (GSE35896)	86.71±0.26	86.95±0.27	86.97±0.26	86.15±0.27	87.03±0.26	86.69±0.28	86.94±0.25	86.97±0.25	**87.16±0.27**
Colorectal (GSE42284)	33.46±0.11	32.47±0.09	33.67±0.11	**34.71±0.13**	31.92±0.11	34.13±0.09	33.55±0.10	32.90±0.12	32.81±0.12
Breast MicroRNA (PAM50)	36.07±0.09	39.15±0.09	34.98±0.05	36.08±0.09	42.32±0.07	35.51±0.07	42.34±0.07	43.20±0.04	**45.11±0.05**
Breast MicroRNA (Histologic)	47.10±0.16	55.52±0.61	57.37±0.19	62.14±0.65	70.00±0.02	60.41±0.17	69.96±0.03	72.05±0.06	**74.04±0.11**
Breast MicroRNA (ER)	58.25±0.35	69.23±0.13	65.95±0.11	64.58±0.18	68.88±0.25	63.61±0.33	68.88±0.25	70.99±0.17	**73.29±0.13**
Breast MicroRNA (PR)	56.50±0.35	63.23±0.15	61.37±0.14	60.92±0.08	63.04±0.21	59.88±0.31	63.05±0.20	64.27±0.13	**65.86±0.10**
Breast MicroRNA (HER2)	58.49±0.04	61.57±0.05	59.51±0.04	54.32±0.05	66.01±0.02	59.93±0.02	65.89±0.02	67.53±0.03	**69.96±0.03**

### Effects of normalization methods

Without using a filter, we can see that the highest clustering accuracy for each dataset is always achieved by one of the normalization methods, among which the maximum norm appears to the best (9 times among 13 settings). Moreover, the smallest standard error of the mean for all settings except 2 cases is achieved by one of normalization methods while using the maximum norm achieves it most often (7 times), indicating that using the maximum norm makes the NMF solutions less sensitive to initial conditions in the NMF iteration algorithm.

We observe that using the maximum norm is the best choice. However, on dataset CNS and GSE4913, using the maximum norm is significantly worse than the Basic NMF that does not employ normalization methods. This does not mean the normalization method using the maximum norm itself goes wrong: there may be some other factors distorting the NMF solutions, based on which the normalization method seems not to be good. One such factors is the effects from irrelevant genes. This is illustrated in a simulation study in S1 File. We speculate that there is similar problem on datasets CNS and GSE4913, so we expect that the filter being intended to remove effects from irrelevant genes improves the clustering accuracy for these two datasets from below.

### Effects of the embedded filter

With the embedded filter, using the maximum norm is even better (11 times) than other normalization methods. It is not the best for both the Colorectal datasets GSE42284 and GSE35896. However, for GSE35896, the difference in mean accuracy between the normalization method using the maximum norm and the best is only 0.44, which is not significant (p-value = 0.3 by a paired two-sided t-test); GSE42284 seems not to be appropriate for NMF clustering analysis as all methods achieve very low accuracy. This suggests that the maximum norm is generally the best choice.

To see the effects of the filter on the best normalization method—using the maximum norm, we extract the last column in Table 2 and compare the results using the filter and those without using the filter in Table C in S1 File. At the significance level 0.01, the differences on three datasets are significant: CNS, Medulloblastoma, and GSE4913, on which it is shown that the filter has an effect of enhancing the performance of the best normalization method. On datasets CNS and GSE4913, the cluster accuracy using the maximum norm together with the embedded filter is much improved compared to that using the maximum norm alone. However, on 5 settings, the standard error of the mean using the filter gets larger than that without using the filter. The reason for this may be, different initial conditions produce different *W*s, based on which the filter selects different gene sets on each run, and this in turn brings a little extra variation. Nevertheless, the filter is important to enhance the performance of the normalization method using the maximum norm when there are many irrelevant genes.

### Effects of the variance-filter

With the variance-filter, using the maximum norm is still the best among other normalization methods. But the variance-filter performs slightly worse than the embedded filter, and it is not so good for CNS, one of the well-investigated core datasets as the embedded filter. One possible reason is that, unlike the embedded filter, the variance-filter is not specially designed for NMF algorithms as it does not make use of *W* returned by an NMF algorithm.

### Overall effects of the normalization method using the maximum norm and the embedded filter

Based on the above observations, there is strong evidence that using the maximum norm together with the embedded filter is superior, and is considered as the proposed post-processing method. To compare the overall effect of both the maximum norm and the filter with the Basic NMF (NMF-Brunet), we summarize the results in Table 1. We observe that the proposed method outperforms the Basic NMF for all the datasets except for GSE42284. 9 out of 13 times, the proposed method achieves smaller standard error of the mean than the Basic NMF.

### Extensive evaluation on core datasets

**Fig 1:**
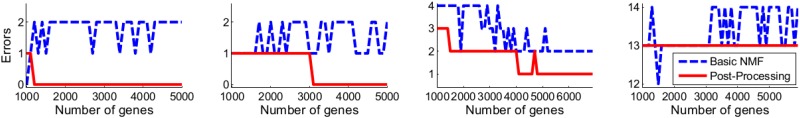
Clustering errors as a function of the number of features (genes) for datasets Leukemia (k = 2), Leukemia (k = 3), CNS (k = 4) and Medulloblastoma (k = 2) respectively. On each of these datasets, the Basic NMF (and the post-processing method using the maximum norm together with the filter) is run on subsets of the full data with 1000 + 100*d* of most highly varying genes (*d* = 0, 1, 2, 3, …). Results are shown as continuous lines for clarity. Clustering error, the number of samples improperly clustered by an algorithm. Here the Basic NMF is the one minimizing a KL divergence in Eq (1).

**Fig 2:**
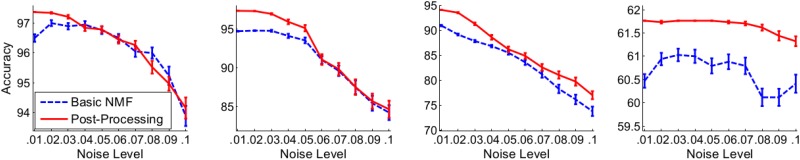
Accuracy as a function of noise levels for datasets Leukemia (k = 2), Leukemia (k = 3), CNS (k = 4) and Medulloblastoma (k = 2) respectively. For each noise level *μ*, NMFs are run 100 times on disturbed matrices. On each of such runs, a disturbed matrix *A*′ is generated by adding independent uniform noises: $A_{i,j}^{\prime }=A_{ij}+\mu *r_{ij}$, where *r*_*ij*_ is a random number generated by a uniform distribution on the interval [0, max], and *max* is the maximum expression in *A*. Plotted is the mean of clustering accuracies from 100 runs together with an error bar representing a standard error of the mean. The post-processing method uses the maximum norm together with the filter.

**Fig 3:**
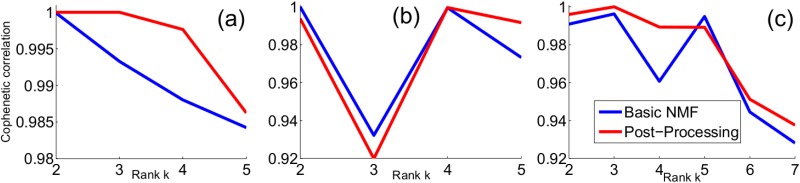
Cophenetic correlation. (a) Leukemia. (b) CNS. (c) Medulloblastoma.

The proposed post-processing method is then evaluated extensively on the core datasets. To show its stability with respect to the number of input genes, we show its performance on these three datasets using a subset of the genes (see Fig 1). To show its robustness to data variations, we add uniform noises onto these three gene expression datasets (See Fig 2). To show the effectiveness of the model selection, we show the cophenetic correlation coefficients for these three datasets (see Fig 3).

#### Leukemia dataset

As far as we know, none of existing methods performs perfectly—for *k* = 2, 100% correctly clustering all 38 samples into 2 clusters; and for *k* = 3, 100% correctly clustering all 38 samples into 3 clusters. There are two errors in the Basic NMF [2] for both *k* = 2 and *k* = 3, which proves to be better than SOM and HC in terms of accuracy or stability [2]. Two alternative methods [5, 7], one (SNMF) explicitly enforcing sparseness in the factorization process, the other one (NMF-Reg) combining both Euclidean distance loss function and generalized Kullback-Leibler divergence loss functions for NMF, produce one error for *k* = 3; and SNMF and NMF-Reg produce zero error, and one error respectively for *k* = 2. Moreover, as reported in a comprehensive evaluation of matrix factorization methods, none of the six matrix factorization methods and two other methods evaluated achieves zero error [20].

In contrast, our method produces zero error using the whole set of genes (Table 1) for both *k* = 2 and *k* = 3. It is also true if we do not use the filter. This is remarkable as two Leukemia samples in this data set have previously been considered to behave as outliers [2] while they are not by this analysis. It is more interesting to observe that our method consistently produces zero error even with just the 1,200 (for *k* = 2) or 3,100 (for *k* = 3) most highly varying genes (Fig 1). Moreover, our method is still better than the Basic NMF when noise is added (Fig 2).

The cophenetic correlation coefficients (see Fig 3(a)) achieve the maximum 1 for both *k* = 2 and *k* = 3, suggesting that there are two subtypes or three subtypes—this is consistent with what is known about the subtypes of this cancer.

#### CNS and Medulloblastoma datasets

On the dataset CNS, SNMF [5] can produce similar clustering accuracy as our method while NMF-Reg [7] produces a worse accuracy of 94.1%. On the Medulloblastoma dataset NMF-Reg [7] can achieve a clustering accuracy of 73.4% which is better than our method while the result for SNMF is not shown explicitly in [5]. On both datasets, our method consistently produces a better clustering than the Basic NMF using the whole set of genes (Table 1) or subsets of genes (Fig 1), and remains better when noise is added (Fig 2).

The cophenetic correlation coefficients (see Fig 3(b)) achieve the maximum for *k* = 4, suggesting that there are four subtypes—this is consistent with the known 4 subtypes. The cophenetic correlation coefficients produced by our method (see Fig 3(b)) are more informative than SNMF: the difference between cophenetic correlation coefficients produced by SNMF for two adjacent values of *k* is small [5] while our method produces a bigger gap; the Basic NMF gives *k* = 2 or *k* = 4 and the Basic NMF for *k* = 2 produces two clusters—one is {9 malignant gliomas, 4 normals, and 1 classic medulloblastomas}, the other is the rest of the samples—this is not reasonable as malignant glioma should be very different from normal.

The cophenetic correlation coefficients (see Fig 3(c)) achieve the maximum for *k* = 3, suggesting that there are three subtypes. Unlike Basic NMF and SNMF that the cophenetic correlation coefficients achieve the maximum for both *k* = 3 or *k* = 5, our method produces a unique peak at *k* = 3. Based on this observation and the poor clustering accuracies, here we note the same caveat as [5] about the sample assignments, given the fact that the pathogenesis of these tumors is not well understood and desmoplastic medulloblastoma diagnosis is highly subjective.

## Discussion

After observing the superior performance of the proposed post-processing method, we try to provide some interpretations on the implicit assumptions that it may rely on. Moreover, we would like to discuss two scenarios, in which the proposed method does not seem to work very well.

### Interpretations

Although the filter in Algorithm 1 is not our main focus in this paper, its justification has been discussed when it is introduced. The unsolved problem is to find a better threshold automatically adapted to the data than the current default setting (*T* = 0.5), which is left as future work. In the following, we shall only focus on the justifications for the normalization by the maximum norm.

#### Memberships of genes in metagenes

One interpretation relates to the concept of a metagene. Recall that a metagene is a positive linear combination of the genes [2], and the *j*-th column of *W* defines a metagene, with entry *w*_*ij*_ the coefficient of gene *i* in metagene *j*. Here we provide a different interpretation of metagenes.

For a typical cancer type, there are some specific activities that control the genes’ expression levels. Some genes are involved in these activities fully (always present with a membership of 1) while others partly (not always present with a membership of less than 1; for example, the pathway including a gene is not always activated). The genes’ expression levels controlled by these activities are proportional to their memberships in these activities. Here we specify the meaning of a metagene as a fuzzy collection of genes, and the coefficient *w*_*ij*_ as the membership of gene *i* in metagene *j*. These activities are reflected in the metagene’s expression that can be explained as the strength that the metagene has in controlling genes’ expression levels. Therefore a metagene can then represent a typical cancer type.

Consequently, the gene expression levels across all samples for each gene *i* (represented by *A*_*i*,:_) can thus be approximated by a linear combination of rows of *H* (metagenes’ expression across all samples, represented by *H*_*j*,:_), and the combination coefficients are specified by the memberships of *i*-th gene in metagenes (*i*-th row of *W*), *i.e.*, *A*_*i*,:_ ∼ ∑_*j*_ *w*_*i*,*j*_ *H*_*j*,:_. In other words, because of the membership *w*_*i*,*j*_ of gene *i* in metagene *j*, the *j*-th metagene’s expression *H*_*j*,:_ will contribute to gene *i* with an amount of *w*_*i*,*j*_ *H*_*j*,:_; and the gene *i*’s expression is accumulated by all such contributions.

While membership is never greater than one, the entries in *W*_*j*_—the *j*-th column of a solution *W* from an NMF algorithm—are not necessarily smaller than or equal to 1. Thus it needs to be normalized. In the proposed method, *W*_*j*_ is divided by its maximum value (the maximum norm of *W*_*j*_) to produce $W_{j}^{\prime }$ for each *j*, and *H*′ is obtained by multiplying each row of *H* by the maximum value of the corresponding column of *W*. This normalization method has the advantage that it restricts all entries in $W_{j}^{\prime }$ to be smaller than or equal to one—this fits our interpretation of metagenes and memberships. There is an implicit assumption underlying this specific normalization method that each metagene must contain at least one gene for sure (the membership is one, *i.e.*, one or more genes are always turned on for some pathways involved in a typical cancer type). Although it is not ideal that the proposed method depends on this mild assumption, other normalization methods mentioned before are less desirable: divided by the p-norm (p = 1, 2, 3) of *W*_*j*_ makes the entries in $W_{j}^{\prime }$ too small while using quantiles makes some of them greater than one.

It can be imagined that the proposed method will suffer from some problems if this assumption does not hold in some scenarios, for which other normalization methods may be appropriate. However empirical evidence in Table 2 does not support other normalization methods.

### Basic NMF minimizing Euclidian distance

In the previous sections, we have shown that the proposed post-processing method works very well compared to the Basic NMF algorithm that minimizes a generalized Kullback-Leibler divergence functional corresponding to a likelihood function with a Poisson noise or a scaled Poisson noise. It is natural to ask whether the proposed method improves other Basic NMF algorithms, for example, NMF-EU [4] that minimizes a Euclidian distance corresponding to a likelihood function with Gaussian noise. Some studies can be found in S1 File. Generally speaking, if the Basic NMF is not derived from a correct noise model, the discovered *W* and *H* will be distorted; and as a result, the post-precessing method based on a distorted output may not achieve its best performance.

### Evaluation of unsupervised methods using datasets designed for supervised algorithms

Some expression datasets are good for the task of classification but not for clustering—if only a small fraction of genes (signature genes) in a dataset differ between different types of tumors while the majority of genes differ between levels of another categorical feature (*e.g.*, gender). In such cases, the clusters discovered by NMF may not be consistent with the cancer types, and the evaluation based on the cancer types will be problematic. This problem can be seen from the evaluation of the proposed method on 11 datasets which were collected for evaluating classification methods [21]: the proposed method does not show consistent improvement over the Basic NMF. Details can be found in S1 File. This problem is illustrated further in a simulation study in S1 File.

## Conclusion

We have investigated the effects of different normalization methods over a Basic NMF (NMF-Brunet) that does not employ normalization methods from [2] on 9 datasets with 13 settings. We have observed that using the maximum norm is the best choice. We have also provided an interpretation to justify the use of the maximum norm.

On the same three datasets that were commonly used by [2, 5–7], we have shown that the proposed post-processing method using the maximum norm together with a filter has important advantages over the Basic NMF: it is less sensitive to *a priori* selection of genes or initial conditions, and more robust to data variations. We have also demonstrated the improved clustering performance of the proposed method on three other gene expression datasets and one additional type of data—MicroRNA expression for Breast cancer.

Given such promising results in clustering accuracy improvement and automatic selection of number of cancer types, this method of using the maximum norm (infinity norm) to normalize NMF solutions should not be ignored as it had been for clustering analysis using NMF techniques, although it is not perfect in itself. One obvious limit of the proposed method is that it depends on the Basic NMF: it requires that the Basic NMF is derived from a correct noise model; otherwise, it may not achieve its best performance. Another imaginable limit is that our assumption about the memberships of genes in a metagene may be violated: none of the memberships is one. Moreover, the filter in Algorithm 1 needs to be investigated further: there may exist a better threshold adapted to the data than the current default setting, in order to improve the performance of the normalization method using the maximum norm.

## Supporting Information

S1 File Supplementary Material. It contains a simulation study, evaluation of unsupervised methods using datasets designed for supervised, effects of the filter on all datasets in the main text, and Basic NMF minimizing Euclidian distance algorithms.
(PDF)
