# Supplementary material for: Impact of the Choice of Normalization Method on Molecular Cancer Class Discovery Using Nonnegative Matrix Factorization
Source: PLoS One. 2016 Oct 14;11(10):e0164880. doi: 10.1371/journal.pone.0164880 (PMC5065197; doi:10.1371/journal.pone.0164880)
Supplement: S1 File — It contains a simulation study, evaluation of unsupervised methods using datasets designed for supervised, effects of the filter on all datasets in the main text, and Basic NMF minimizing Euclidian distance algorithms. (PDF) [file pone.0164880.s001.pdf]

Supplementary material to "Impact of the choice of normalization method on molecular cancer class discovery using nonnegative matrix factorization"

Haixuan Yang, and Cathal Seoighe

October 5, 2016

Contents

1 A simulation study **2**
1.1 Data A 2
1.2 Data A+ 3
1.3 Data B 3
1.4 Results 5
1.4.1 Meaningless results on A+ — an answer to Question 1 5
1.4.2 Effects of normalization methods on A and B— an answer to Question 3 6
1.4.3 Effects of irrelevant genes and the filter — an answer to Question 2 . 6

2 Evaluation of unsupervised methods using datasets designed for supervised algorithms **7**

3 Effects of the filter on all datasets in the main text **9**

4 Basic NMF minimizing Euclidian distance **10**

1 A simulation study

Here we conduct a simulation study, by which we try to find evidence to support answers to the following questions:

Question 1: What will happen if there is only a small fraction of genes (signature genes) in a dataset differ between different types of tumors while more genes differ between levels of another categorical feature?

Question 2: What is the effect of irrelevant genes and the filter of removing irrelevant genes?

Question 3: What is the effect of a normalization method?

We design an artificial dataset containing 60 samples consisting of two classes I and II, representing two cancer types. 20 samples belong to class I, and the remaining 40 belong to class II. Here we consider three cases (A, A+, and B). To answer Question 2, we consider a case (data A) that contains 100 signature genes of the cancer types and 1000 irrelevant genes; also we consider a case (data B) that contains contains 100 signature genes of the cancer types and 50 irrelevant genes. To answer Question 1, we consider a case (data A+), in which some more signature genes discriminating another category are added to A. Then we compare the effects of different normalization methods to answer Question 3. Note that here we use NMF-Brunet as the Basic NMF that does not normalize the NMF solutions and it does not use the filter in Algorithm 1 in the main text. Because a Poisson noise is assumed underlying the NMF-Brunet, we shall generate data using a Poisson noise. The data will be repeatedly generated 100 times for evaluations.

1.1 Data A

In the first case, we try to make the data that contains 100 signature genes that can discriminate the two classes. Moreover, it contains another 1000 irrelevant genes that do not discriminate the two classes, and the data is generated by a Poisson noise. For this, we expect our post-processing method is better than the Basic NMF. We generate A 100 times by the following steps:

1. W(i,j)=1 (i=1,2,. . . ,50; j=1); W(i,j)=1 (i=51,52,. . . ,100; j=2); W(i,j)=0.7 (i=101, 102, 1100; j=1, 2); W(i,j) is independently drawn from a uniform distribution over the interval [0,1] otherwise.
2. H(i,j)=100 (i=1; j=1,2,. . . ,20); H(i,j)=100 (i=2; j=21,22,. . . ,60); H(i,j) is independently drawn from a uniform distribution over the interval [0, 92.5] otherwise.
3. A0=W*H.
4. A=Poisson(A0).

Among 100 realizations of these matrices, one snapshot of the matrices W, H, A0=W*H, and A=Poisson(A0) is shown in Fig. 1.

1.2 Data A+

In the second case, there are two other classes III and IV (e.g., Male and Female), and there are more signature genes discriminating classes III and IV than genes discriminating classes I and II. It is designed that half of the samples in class I and half of the samples in class II belong to class III while the remaining belongs to class IV. Based on A0, we add 200 signature genes that discriminate classes III and IV to generate A0+, and A+ is generated by adding a Poisson noise (A+=Poisson(A0+)). In such cases, the clusters discovered by NMF may not be consistent with the cancer types (I and II), and the evaluation based on the cancer types will be problematic. Among 100 realizations of these matrices, one realization of the matrices A0+, and A+=Poisson(A0+) are shown in Fig. 1.

1.3 Data B

In the third case, similar to data A, we try to make the data that contains 100 signature genes that can discriminate the two classes. Different from data A, it contains only 50 additional irrelevant genes that do not discriminate the two classes, and the data is generated by a Poisson noise. For this, we expect our post-processing method is better than the Basic NMF. Among 100 realizations of these matrices, one realization of the matrices W, H, B0=W*H, and B=Poisson(B0) is shown in Fig. 2.

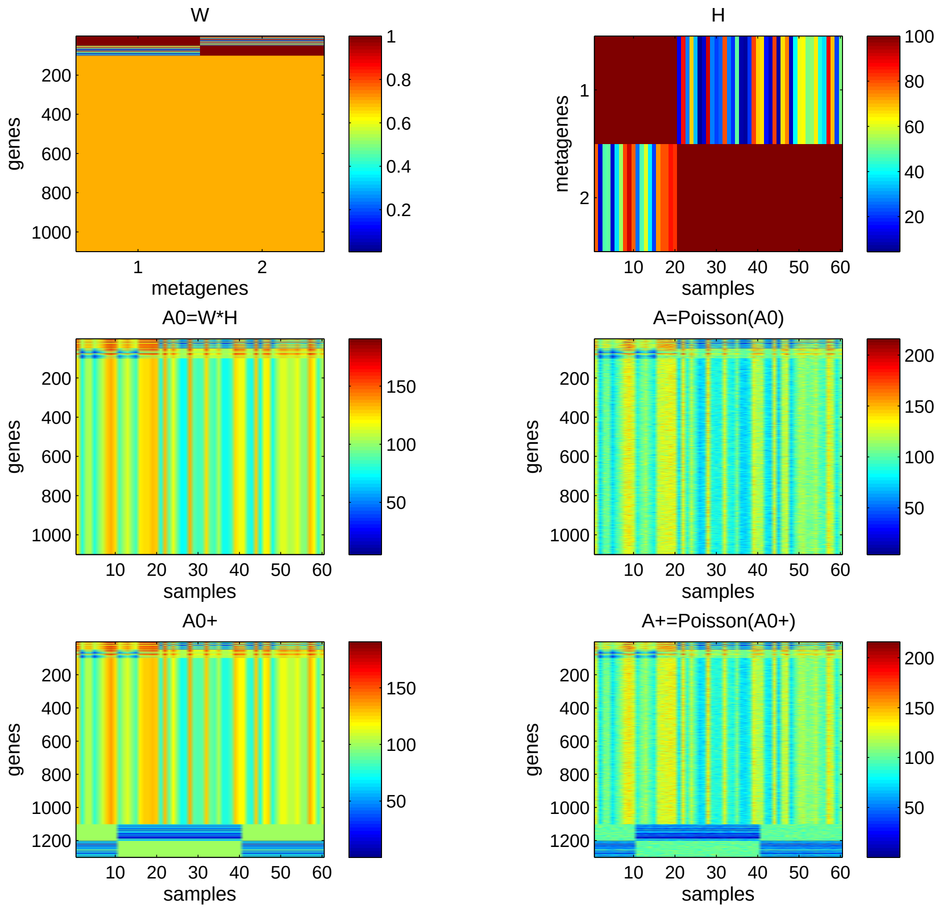

Figure 1: Toy data A and A+.

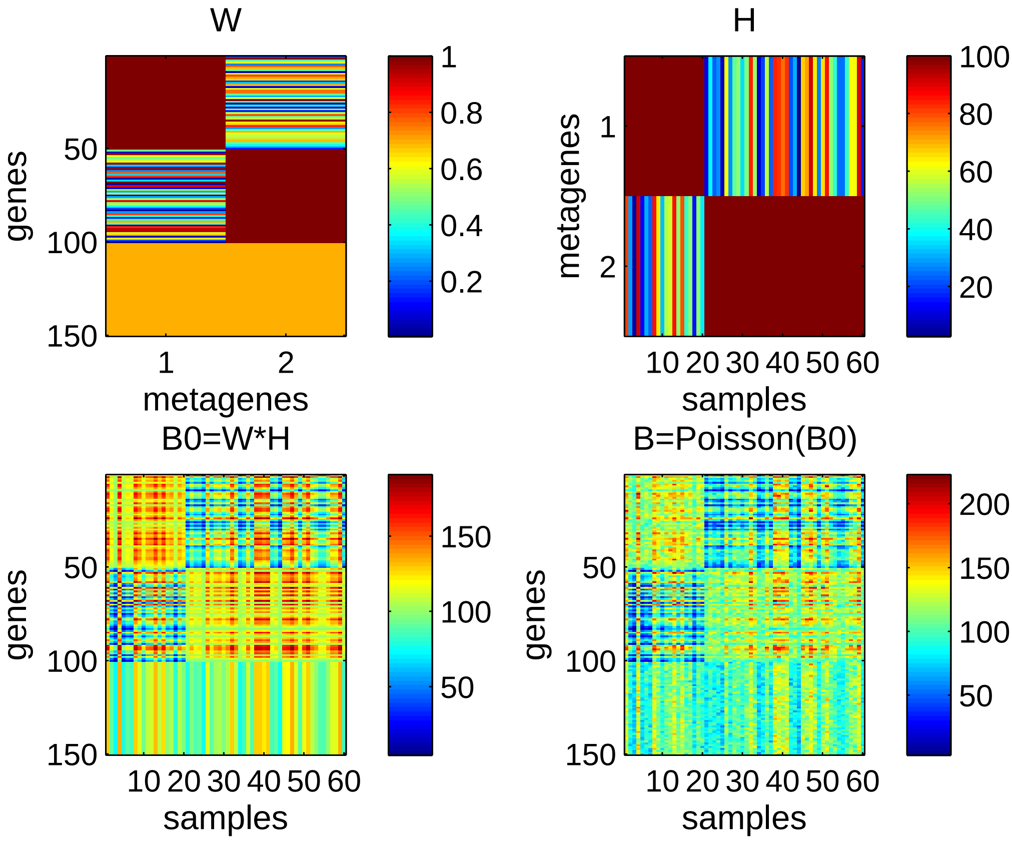

Figure 2: Toy data B.

1.4 Results

We show the results in Table A for both settings of using the filter or not. We used the default T=0.5. We performed 100 independent trials and computed the mean accuracy and the standard error of the mean. The clustering accuracy results are evaluated by classes I and II. In each of the 100 runs, A+ is re-generated by a Poisson noise based on a re-generated A0+, and H and W are randomly initialized. A corresponds to the 1100 rows of A+ without adding 200 signature genes for classes III and IV.

1.4.1 Meaningless results on A+ — an answer to Question 1

On the data A+, all methods do not work well for the purpose of grouping samples by cancer types. As we expected, the post-processing method cannot improve over the Basic NMF. The reason for this, is that the data tends to prefer clusters by classes III and IV (genders) to clusters by classes I and II (cancer types). While it is not difficult to select marker genes for cancer types given labels from the data A+ in a supervised learning algorithm, it is problematic to find meaningful clusters for cancer types in an unsupervised setting from a data having a lot of nuisance genes. Consequently the evaluation of performances for clustering

Datasets	Unnormalized	0.95q	0.75q	0.5q	SD	1-norm	2-norm	3-norm	max
**With Filter** (Filter=1 in Algorithm 1)									
A	95.30±4.52	99.05±2.12	98.57±2.72	98.45±2.86	**99.37±1.27**	98.53±2.50	98.47±2.48	98.75±2.41	**99.17±1.92**
A+	50.00±0.00	50.00±0.00	50.00±0.00	50.00±0.00	50.00±0.00	50.00±0.00	50.00±0.00	50.00±0.00	50.00±0.00
B	95.52±4.60	99.02 ± 1.94	99.15±1.75	90.05±7.63	99.27±1.76	97.85±3.48	98.82±2.25	98.78±2.32	98.55±3.15
**Without Filter** (Filter=0 in Algorithm 1)									
Datasets	**NMF-Brunet**	0.95q	0.75q	0.5q	SD	1-norm	2-norm	3-norm	max
A	92.95±6.10	96.57±4.89	97.55±3.80	97.65±3.77	**99.33±1.28**	97.63±3.78	97.73±3.64	97.73±3.64	**98.55±2.79**
A+	50.00±0.00	50.00±0.00	50.00±0.00	50.00±0.00	50.00±0.00	50.00±0.00	50.00±0.00	50.00±0.00	50.00±0.00
B	95.25±4.40	**99.17**±1.68	**99.20**±1.68	98.47±2.58	98.80±2.19	98.60±2.42	98.82±2.15	98.90±2.10	**99.07±1.71**

Table A: Comparison of different normalization methods in term of clustering accuracy in percentage. Reported is the mean of clustering accuracies from 100 runs of Basic NMF together with the standard error of the mean.

algorithms on datasets like A+ is meaningless. We speculate that there are some similar situations as A+ in some datasets whose original research is mainly focused on classification problems.

1.4.2 Effects of normalization methods on A and B— an answer to Question 3

On the data A, the Basic NMF works reasonably well. Without using the filter, using the maximum norm appears to be the second best while the normalization method SD is the best (the mean clustering accuracy is significantly different from that produced by the maximum norm, p-value=0.0083). With the filter, the normalization method SD appears to the best, but the mean clustering accuracy is not significantly different from that produced by the maximum norm (p-value=0.3656). Thus we conclude that using the maximum norm is a good choice for both setting of using the filter or not. It is surprising to see that the normalization method SD is excellent on the toy data A, and we could not explain why this happens. Nevertheless, it is not so great on the real data.

On the data B, without the filter, the normalization method 0.75q appears to the best, but the mean clustering accuracy is not significantly different from that produced by the maximum norm (p-value= 0.1172). With the filter, the situation is changed: see the discussion below.

1.4.3 Effects of irrelevant genes and the filter — an answer to Question 2

On the data A, we can see that, the filter improves all the normalization methods. This result may come from the effects from irrelevant genes (1000 genes that do not discriminate the two classes in this example). With the filter being intended to remove effects from irrelevant genes, such effects are expected to be removed to some extent and the clustering accuracy is improved. However, on the data B, the filter does not work any more. This is probably because less than half of the genes are irrelevant in B while the filter with the default setting removes half of the genes. This means that the current default setting of the threshold is not optimal, and this justifies the need of a further investigation on the filter in the future work. This is why we state in the end of the main text that "the filter in Algorithm 1 needs to be investigated further: there may exist a better threshold adapted to the data than the current default setting, in order to improve the performance of the normalization method using the maximum norm."

2 Evaluation of unsupervised methods using datasets designed for supervised algorithms

We download 11 gene expression datasets from http://www.gems-system.org/. They were collected for evaluating classification methods and their description can be found (Statnikov *et al.*, 2005). The preprocessing method of these datasets was summarized in Table 1 in (Liu *et al.*, 2008). The cluster accuracy for both the Basic NMF and the Post-Processing method is reported in Fig. 3).

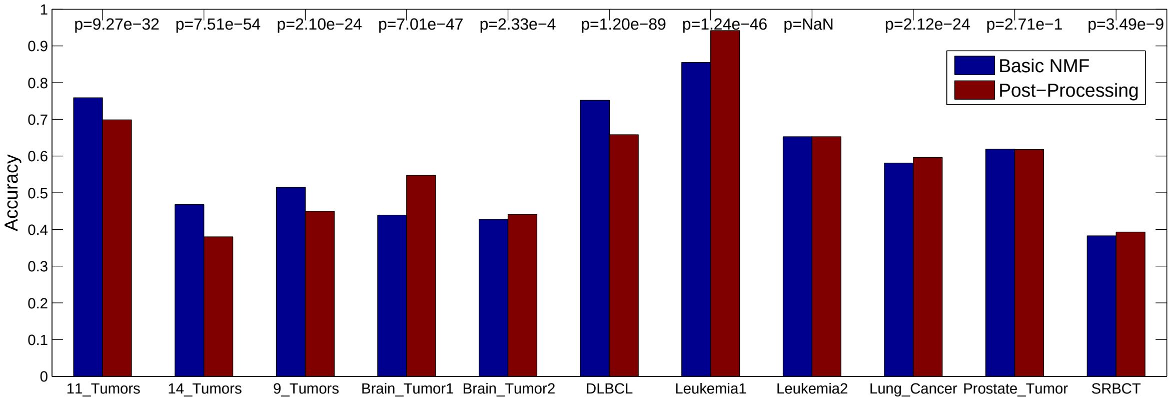

Figure 3: Clustering accuracy for 11 datasets. Reported is the mean of clustering accuracies from 100 runs of Basic NMF together with the p-value produced by a paired two-sided t-test. The p-values are shown on the top of the figure.

Dataset	Reference	Original research focus
11_Tumors	Su *et al.* (2001)	Classification
14_Tumors	Ramaswamy *et al.* (2001)	Classification
9_Tumors	Staunton *et al.* (2001)	Classification
Brain_Tumor1*	Pomeroy *et al.* (2002)	Classification and Clustering
Brain_Tumor2	Nutt *et al.* (2003)	Classification
DLBCL	Shipp *et al.* (2002)	Classification
Leukemia1*	Golub *et al.* (1999)	Classification and Clustering
Leukemia2*	Armstrong *et al.* (2002)	Classification and Clustering
Lung_Cancer*	Bhattacharjee *et al.* (2001)	Classification and Clustering
Prostate_Tumor*	Singh *et al.* (2002)	Classification and Clustering
SRBCT*	Khan *et al.* (2001)	Classification and Clustering

Table B: Datasets and their original research problems. Those datasets that were used for clustering analysis in the original papers are marked a *, indicating that they are more appropriate for the purpose of evaluation of clustering algorithms. Note that in (Shipp *et al.*, 2002), they did not do clustering analysis for DLBCL outcome although they did some initial clustering analysis. There is a similar situation in Su *et al.* (2001). Note datasets Leukemia1 and Brain_Tumor1 are supersets of the datasets used in the main text.

The proposed method does not show consistent improvement over the Basic NMF. In the main text, we have speculated that some expression datasets are good for the task of classification

but not for clustering – if only a small fraction of genes (signature genes) in a dataset differ between different types of tumors while the majority of genes differ between levels of another categorical feature (*e.g.*, gender). In such cases, the clusters discovered by NMF may not be consistent with the cancer types, and the evaluation based on the cancer types will be problematic. This problem is illustrated further by a simulation study. Nevertheless, it is still mysterious to us that why the proposed method looks much worse than the Basic NMF for some classification datasets even if the evaluation of performances for clustering algorithms is meaningless. We have not been able to design a simulation study to explain this yet.

Although all these datasets were used to evaluate classification methods in (Statnikov *et al.*, 2005), some of them were also used for clustering purpose in their original papers (see Table B), namely Brain_Tumor1, Leukemia1, Leukemia2, Lung_Cancer, SRBCT, and Prostate_Tumor. These datasets are thus more appropriate for the purpose of evaluation of clustering algorithms. Among them, only on Prostate_Tumor dataset, the proposed method performs slightly worse (but not significantly as the p-value is 0.271) than the Basic NMF. Therefore, from this set of evaluation, there is no evidence against the proposed method.

3 Effects of the filter on all datasets in the main text

To see the effects of the filter on the best normalization method – using the maximum norm, we compare the effects on all datasets in Table C.

Datasets	#Types	#Samples	#Genes	max (without filter)	max (with filter)	p-values
**Core Datasets**						
Leukemia	2	38	5,000	**100±0**	**100±0**	NaN
Leukemia	3	38	5,000	**100±0**	**100±0**	NaN
CNS	4	34	6,881	91.26±0.07	**96.88±0.07**	2.51e-079
Medulloblastoma	2	34	5,893	61.53±0.08	**61.76±0**	4.16e-003
**Extended Datasets**						
Breast Recurrence (GSE4913)	2	50	5,000	50.00±0	**62.48±0.28**	4.23e-067
Pancreas (GSE46385)	5	47	5,000	50.79 ±0.57	**51.57±0.60**	0.32
Colorectal (GSE35896)	2	62	5,000	**88.92±0.29**	88.00±0.32	0.025
Colorectal (GSE42284)	4	188	5,000	**32.46±0.14**	32.13±0.12	0.082
Breast_MicroRNA (PAM50)	5	503	1,007	**45.12±0.02**	**45.12±0.02**	1
Breast MicroRNA (Histologic)	4	643	1,017	74.11±0.11	**74.24±0.13**	0.46
Breast MicroRNA (ER)	2	677	1,022	73.24±0.11	**73.34±0.11**	0.51
Breast MicroRNA (PR)	2	674	1,022	65.85±0.10	**65.93±0.11**	0.56
Breast MicroRNA (HER2)	2	487	1,009	69.97±0.03	**69.99±0.03**	0.63

Table C: Performance comparison in term of clustering accuracy in percentage for the effects of the filter on the best normalization method – using the maximum norm. Reported is the mean of clustering accuracies from 100 runs of Basic NMF together with the standard error of the mean. Also reported is the p-value produced by a paired two-sided t-test. Note that the proposed method is using 'max' normalization and using the filter.

4 Basic NMF minimizing Euclidian distance

The underlying assumption for NMF-EU is that $A_{i,j}$ is an observation from a normal distribution with mean $(WH)_{i,j}$ and a constant variance σ^2, and W and H are model parameters.

Here, if the Basic NMF is the one minimizing Euclidian distance, the results of the post-processing method are impressive in terms of clustering stability (see Fig. 4), as we expected. But it is not so in terms of clustering accuracy. For this, an explanation is that the KL divergence functional is better suited as a loss function for the matrix factorization for the underlying problems, i.e., the assumption of a Poisson noise is more reasonable than a Gaussian noise for the nonnegative expression data.

The improper use of Gaussian noise can be verified qualitatively: $A_{i,j}$ may be negative if it is from a normal distribution, but it never is. Or quantitatively this can be verified by showing the residuals $A - WH$ produced by NMF-EU are not normally distributed while the model assumes that they should be. The Q-Q plots of the residuals $A - WH$ in Fig. 5 strongly contradict the normality of the residuals as they are not a straight line for all these datasets with four settings.

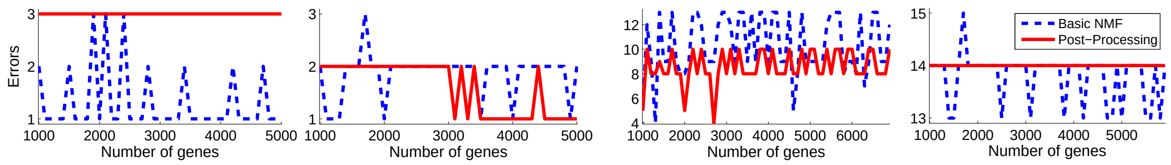

Figure 4: Clustering errors as a function of the number of features (genes) for datasets Leukemia (k=2), Leukemia (k=3), CNS (k=4) and Medulloblastoma (k=2) respectively. On each of these datasets, the Basic NMF (and the Post-Processing method) is run on subsets of the full data with $1000 + 100d$ of most highly varying genes ($d = 0, 1, 2, 3, ...$). Results are shown as continuous lines for clarity. Clustering error, the number of samples improperly clustered by an algorithm. Here the Basic NMF is the one minimizing a Euclidian distance (NMF-EU).

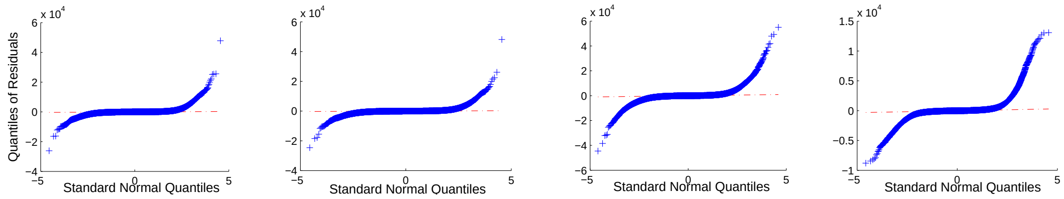

Figure 5: QQ Plots of Residuals ($A - WH$ produced by NMF-EU) versus Standard Normal for Leukemia (k=2), Leukemia (k=3), CNS (k=4) and Medulloblastoma (k=2) respectively. These plots do not support the assumption of normal residuals underlying NMF-EU.

References

Armstrong, S. A., Staunton, J. E., Silverman, L. B., Pieters, R., den Boer, M. L., Minden, M. D., Sallan, S. E., Lander, E. S., Golub, T. R., and Korsmeyer, S. J. (2002). Mll translocations specify a distinct gene expression profile that distinguishes a unique leukemia. *Nature genetics*, **30**(1), 41–47.

Bhattacharjee, A., Richards, W. G., Staunton, J., Li, C., Monti, S., Vasa, P., Ladd, C., Beheshti, J., Bueno, R., Gillette, M., *et al.* (2001). Classification of human lung carcinomas by mrna expression profiling reveals distinct adenocarcinoma subclasses. *Proceedings of the National Academy of Sciences*, **98**(24), 13790–13795.

Brunet, J.-P. *et al.* (2004). Metagenes and molecular pattern discovery using matrix factorization. *Proceedings of the National Academy of Sciences*, **101**(12), 4164–4169.

Golub, T. R., Slonim, D. K., Tamayo, P., Huard, C., Gaasenbeek, M., Mesirov, J. P., Coller, H., Loh, M. L., Downing, J. R., Caligiuri, M. A., Bloomfield, C. D., and Lander, E. S. (1999). Molecular classification of cancer: Class discovery and class prediction by gene expression monitoring. *Science*, **286**(5439), 531–537.

Khan, J., Wei, J. S., Ringner, M., Saal, L. H., Ladanyi, M., Westermann, F., Berthold, F., Schwab, M., Antonescu, C. R., Peterson, C., *et al.* (2001). Classification and diagnostic prediction of cancers using gene expression profiling and artificial neural networks. *Nature medicine*, **7**(6), 673–679.

Liu, W., Yuan, K., and Ye, D. (2008). On α-divergence based nonnegative matrix factorization for clustering cancer gene expression data. *Artificial intelligence in medicine*, **44**(1), 1–5.

Nutt, C. L., Mani, D., Betensky, R. A., Tamayo, P., Cairncross, J. G., Ladd, C., Pohl, U., Hartmann, C., McLaughlin, M. E., Batchelor, T. T., *et al.* (2003). Gene expression-based classification of malignant gliomas correlates better with survival than histological classification. *Cancer research*, **63**(7), 1602–1607.

Pomeroy, S. L., Tamayo, P., Gaasenbeek, M., Sturla, L. M., Angelo, M., McLaughlin, M. E., Kim, J. Y., Goumnerova, L. C., Black, P. M., Lau, C., *et al.* (2002). Prediction of central nervous system embryonal tumour outcome based on gene expression. *Nature*, **415**(6870), 436–442.

Ramaswamy, S., Tamayo, P., Rifkin, R., Mukherjee, S., Yeang, C.-H., Angelo, M., Ladd, C., Reich, M., Latulippe, E., Mesirov, J. P., *et al.* (2001). Multiclass cancer diagnosis using tumor gene expression signatures. *Proceedings of the National Academy of Sciences*, **98**(26), 15149–15154.

Shipp, M. A., Ross, K. N., Tamayo, P., Weng, A. P., Kutok, J. L., Aguiar, R. C., Gaasenbeek, M., Angelo, M., Reich, M., Pinkus, G. S., *et al.* (2002). Diffuse large b-cell lymphoma outcome prediction by gene-expression profiling and supervised machine learning. *Nature medicine*, **8**(1), 68–74.

Singh, D., Febbo, P. G., Ross, K., Jackson, D. G., Manola, J., Ladd, C., Tamayo, P., Renshaw, A. A., D'Amico, A. V., Richie, J. P., *et al.* (2002). Gene expression correlates of clinical prostate cancer behavior. *Cancer cell*, **1**(2), 203–209.

Statnikov, A., Aliferis, C. F., Tsamardinos, I., Hardin, D., and Levy, S. (2005). A comprehensive evaluation of multicategory classification methods for microarray gene expression cancer diagnosis. *Bioinformatics*, **21**(5), 631–643.

Staunton, J. E., Slonim, D. K., Coller, H. A., Tamayo, P., Angelo, M. J., Park, J., Scherf, U., Lee, J. K., Reinhold, W. O., Weinstein, J. N., *et al.* (2001). Chemosensitivity prediction by transcriptional profiling. *Proceedings of the National Academy of Sciences*, **98**(19), 10787–10792.

Su, A. I., Welsh, J. B., Sapinoso, L. M., Kern, S. G., Dimitrov, P., Lapp, H., Schultz, P. G., Powell, S. M., Moskaluk, C. A., Frierson, H. F., *et al.* (2001). Molecular classification of human carcinomas by use of gene expression signatures. *Cancer research*, **61**(20), 7388–7393.
